# Histological interpretation of differentiated vulvar intraepithelial neoplasia (dVIN) remains challenging—observations from a bi-national ring-study

**DOI:** 10.1007/s00428-021-03070-0

**Published:** 2021-03-08

**Authors:** Shatavisha Dasgupta, Elf de Jonge, Mieke R. Van Bockstal, Luthy S. M. Wong-Alcala, Suzanne Wilhelmus, Lex A. C. F. Makkus, Katrien Schelfout, Koen K. Van de Vijver, Sander Smits, Etienne Marbaix, Senada Koljenović, Folkert J. van Kemenade, Patricia C. Ewing-Graham

**Affiliations:** 1 Department of Pathology, Erasmus MC, University Medical Centre, Postbus 2040, Be-building, 3000CA, Rotterdam, The Netherlands; 2 Department of Pathology, Groene Hart Ziekenhuis, Gouda, The Netherlands; 3 Department of Pathology, Cliniques Universitaires Saint-Luc Bruxelles, Brussels, Belgium; 4 Department of Pathology, Pathologisch en Cytologisch laboratorium, Breda, The Netherlands; 5 Department of Pathology, Pathan B.V., Laboratory for Pathology, Rotterdam, The Netherlands; 6 Laboratory for Pathology, PAL Dordrecht, Dordrecht, The Netherlands; 7 Department of Pathology, Bravis Ziekenhuis, Bergen op Zoom, The Netherlands; 8 Department of Pathology, Ziekenhuis Geel, Geel, Belgium; 9 Department of Pathology, Cancer Research Institute Ghent, Ghent University Hospital, Ghent, Belgium; 10 Department of Pathology, Antwerp University, Antwerp, Belgium

**Keywords:** Lower genital tract, Carcinoma in situ, Vulvar diseases, Histology, Observer variation

## Abstract

Differentiated vulvar intraepithelial neoplasia (dVIN) is a premalignant lesion that is known to progress rapidly to invasive carcinoma. Accurate histological diagnosis is therefore crucial to allow appropriate treatment. To identify reliable diagnostic features, we evaluated the inter-observer agreement in the histological assessment of dVIN, among a bi-national, multi-institutional group of pathologists. Two investigators from Erasmus MC selected 36 hematoxylin-eosin-stained glass slides of dVIN and no-dysplasia, and prepared a list of 15 histological features of dVIN. Nine participating pathologists (i) diagnosed each slide as dVIN or no-dysplasia, (ii) indicated which features they used for the diagnosis, and (iii) rated these features in terms of their diagnostic usefulness. Diagnoses rendered by > 50% participants were taken as the consensus (gold standard). p53-immunohistochemistry (IHC) was performed for all cases, and the expression patterns were correlated with the consensus diagnoses. Kappa (*ĸ*)-statistics were computed to measure inter-observer agreements, and concordance of the p53-IHC patterns with the consensus diagnoses. For the diagnosis of dVIN, overall agreement was moderate (*ĸ* = 0.42), and pair-wise agreements ranged from slight (*ĸ* = 0.10) to substantial (*ĸ* = 0.73). Based on the levels of agreement and ratings of usefulness, the most helpful diagnostic features were parakeratosis, cobblestone appearance, chromatin abnormality, angulated nuclei, atypia discernable under × 100, and altered cellular alignment. p53-IHC patterns showed substantial concordance (*ĸ* = 0.67) with the consensus diagnoses. Histological interpretation of dVIN remains challenging with suboptimal inter-observer agreement. We identified the histological features that may facilitate the diagnosis of dVIN. For cases with a histological suspicion of dVIN, consensus-based pathological evaluation may improve the reliability of the diagnosis.

**Supplementary Information:**

The online version contains supplementary material available at 10.1007/s00428-021-03070-0.

## Introduction

Differentiated vulvar intraepithelial neoplasia (dVIN) is the immediate precursor of human papillomavirus (HPV)–independent vulvar squamous cell carcinoma (VSCC), and is postulated to develop on the background of chronic dermatoses, driven by *TP53* mutations [1–4]. Recent literature suggests that dVIN has an accelerated rate of progression to VSCC (median interval: 41.4 months), and a high recurrence rate [5–7]. In view of this, current treatment guidelines [8, 9] recommend surgical excision of lesions that are histologically diagnosed as dVIN. Evidently, accurate histological diagnosis is crucial to allow appropriate patient management.

On histology, distinguishing dVIN from dermatoses, such as lichen sclerosus (LS), can present a challenge, as dVIN often exhibits subtle atypical features that mimic the reactive changes seen in chronic dermatoses [10–12]. The difficulty of diagnosing dVIN can give rise to diagnostic variability, which has the potential to critically affect treatment decisions [13, 14].

Although the diagnostic difficulty of dVIN has been acknowledged in literature [2–5], there is insufficient data on the inter-observer agreement in the histological assessment. In a previous study, we established the features that helped to reliably distinguish dVIN from LS, and could be interpreted with substantial agreement by pathologists at our center [15]. However, it remains to be determined whether similar level of agreement can be achieved between pathologists from different practice settings.

In the current study, therefore, we evaluated the inter-observer agreement for the diagnosis, and in the interpretation of histological features of dVIN, among a bi-national, multi-institutional group of pathologists. We also assessed the perception of the pathologists regarding the diagnostic usefulness of the histological features. Our aim was to thereby identify reliable diagnostic features that may facilitate the diagnosis of dVIN. In addition, we correlated the immunohistochemical expression patterns of p53 with the consensus histological diagnoses, as this marker is frequently used as an ancillary tool to support the histological diagnosis of dVIN.

## Materials and methods

### Study design

For the purpose of this study, two investigators (SDG and PCEG) identified all vulvar lesions from 2010 to 2013, from the electronic records of the Department of Pathology, Erasmus MC. All of these lesions were from patients who underwent vulvar biopsies or excisions at Erasmus MC. Hematoxylin-eosin (HE)–stained slides of these lesions were retrieved from the archives, and the histology was reviewed by these investigators.

From this series, the investigators selected a set of 36 slides for inclusion in this study. The selection was enriched for lesions regarded as dVIN by the investigators on histology review, since the aim was to evaluate inter-observer agreement in dVIN. Furthermore, to provide a range of challenges to the participants, the selection was prepared in a way to include (i) lesions adjacent to VSCC, as well as standalone lesions, and (ii) lesions with classical histology, which were diagnostically straightforward, as well as lesions where the distinction between dVIN and no-dysplasia could be difficult. The selection did not comprise any slides with invasive carcinoma, as presence of VSCC in the adjacent epithelium can be considered by pathologists as a diagnostic clue for dVIN [14].

Therefore, of the 36 selected slides, 25 contained lesions adjacent to VSCCs, and 11 contained standalone lesions. The investigators had judged 26 (72%) slides as dVIN and 10 (28%) slides as no-dysplasia, comprising 6 lichen sclerosus and 4 non-specific reactive lesions. The investigators perceived 67% of the diagnoses as straightforward and 33% as difficult.

The original diagnoses of these slides, or the diagnoses rendered by the investigators on review were not used for the analyses. For each slide, the diagnosis rendered by > 50% of the participants was taken as the consensus diagnosis/gold standard.

For de-identification, all slides were re-labeled with opaque stickers bearing a random number. No serial sections were prepared. To ensure that all pathologists evaluated identical areas, the regions of interest were marked on the glass slides with red lines.

For all included slides, immunohistochemistry (IHC) was conducted with (i) p16 (E6H4-clone, Ventana), to confirm that the selection did not contain any HPV-related lesion, and with (ii) p53 (Ventana), to correlate with the consensus diagnosis. The IHC protocol is detailed in supplementary document 1. IHC slides were read only by the investigators and were not provided to the participants. IHC was scored and interpreted as described below:

(i) p16-IHC patterns were scored as block-type or non-block-type (patchy), following the guidelines of The Lower Anogenital Squamous Terminology Standardization Project (LAST) [16]. Block-type p16-expression is considered to be indicative of a high-risk HPV-infection [16]. This pattern was not present in any slide, confirming that the selection did not contain any HPV-related lesion.
(ii) p53-IHC patterns were scored as p53-mutant or p53-wild-type, following recent literature [17, 18]. p53-mutant patterns include basal to para-basal/diffuse overexpression, basal overexpression, null-pattern, or cytoplasmic expression, and these have been reported to strongly correlate with the presence of *TP53* mutations [17–19]. Presence of any of these patterns, therefore, can be considered supportive of a histological diagnosis of dVIN.
  p53-wild-type pattern, i.e., scattered, heterogeneous, basal/para-basal expression, is primarily seen in non-dysplastic lesions. However, this pattern has been also occasionally observed in dVIN [15, 20–23]. Hence, a p53-wild-type pattern does not preclude a histological diagnosis of dVIN. p53 patterns observed in our slides are presented in the “Results.”

Next, a list of histological features of dVIN was compiled from previously published literature [13–15, 24–26], and incorporated into an assessment form. These comprised

A. Features of nuclear atypia: (i) atypia discernable under × 100 magnification; (ii) angulated nuclei; (iii) macronucleoli, i.e., nucleoli visible under × 100 magnification; (iv) chromatin abnormality (open or hyperchromatic pattern); (v) multinucleation; (vi) suprabasal mitoses; (vii) atypical mitoses; and (viii) mitotic count >5/5 mm
B. Features of disturbed maturation/architecture: (i) individual cell keratinization; (ii) deep keratinization; (iii) deep squamous eddies, i.e., abortive pearls of keratin; (iv) cobblestone appearance, i.e., combination of premature keratinization and spongiosis; (v) elongated and/or anastomosing rete ridges; (vi) altered cellular alignment; and (vii) parakeratosis.

### Participants

Pathologists who attend the gynecological-pathology working group of the Rotterdam region were invited to participate. HE-stained glass slides were circulated among the participants for histological assessment. Instructions and forms for the assessment (supplementary document 2) were sent to the participants electronically. Clinical information, original diagnoses, or IHC results were not provided. There was no consensus meeting prior to the assessment to determine any diagnostic criteria. To allow the participants to interpret the histological features in light of their own experience, detailed instructions regarding this were not provided. For measuring 5 mm to assess the mitotic count, participants could use an eye-piece graticule, or the field-diameter of the eye-pieces of their microscopes. Since the measure of 5 mm was an arbitrarily chosen cut-off, a rough estimate of this measurement was considered sufficient. The participants were masked from each other’s assessments. Information regarding the nature of practice (academic/non-academic), country of practice, and length of practicing experience was gathered from the participants.

### Histological assessment

Participants were asked to independently examine the areas marked on the slides, and:

(i) Provide a diagnosis as – dVIN or no-dysplasia
(ii) Score the histological features (listed above) as – not present or present, and if present, indicate whether they were useful, or very useful for the diagnosis of dVIN
(iii) Indicate whether the diagnosis was easy or difficult

### Ethics statement

This study was conducted in accordance with the guidelines of the Dutch Federation of Biomedical Scientific Societies (www.federa.org/codes-conduct), which state that no separate ethical approval is required for the use of anonymized residual tissue procured during regular treatment.

### Statistical analysis

Data were analyzed after all participants had completed their assessments, using R Core Team (2020) (Version 4.0.0, https://www.R-project.org/). Histological diagnoses and features were assessed categorically. Inter-observer agreement was measured by computing (i) percentages of agreement – to obtain an absolute measure, and (ii) kappa (*ĸ*) statistics – to obtain a relative measure. Fleiss’ *ĸ* was computed to measure the overall agreement, i.e., agreement among all participants, using packages “irr” and “raters” [27, 28]. Cohen’s *ĸ* was computed to measure the agreement between each participant pair; this resulted in 36 *ĸ*-values for the diagnoses, as well as for each of the 15 histological features. Cohen’s *ĸ* was also used to measure the concordance of the p53-IHC patterns with the consensus diagnoses. Bootstrapping (10,000 runs) was performed to calculate the 95% confidence intervals (CI) of the *ĸ*-values using the package “boot” [29]. *ĸ*-values were interpreted as follows: < 0.20 = slight, 0.21–0.40 = fair, 0.41–0.60 = moderate, 0.61–0.80 = substantial, or 0.81–1.00 = near-perfect agreement. Correlation between categorical variables was measured with chi (*χ*^2^)-squared test; two-sided *p*-value < 0.05 was considered statistically significant. Heat maps and bar charts were constructed to visualize the data.

## Results

### Participants

Nine pathologists participated in this study; 6 practice at 5 non-academic centers in the Netherlands, which handle a high diagnostic case load, and 3 practice at 2 academic centers in Belgium. Lengths of their practice experience ranged from less than 5 years (*n* = 2) to more than 15 years (*n* = 3). All participants routinely read vulvar pathology cases, including dVIN and VSCC, in their practice. The participants have been anonymized and are represented by acronyms (P1–P9), which do not correspond to their order in the author list.

### Histological assessment

**Fig. 1:**
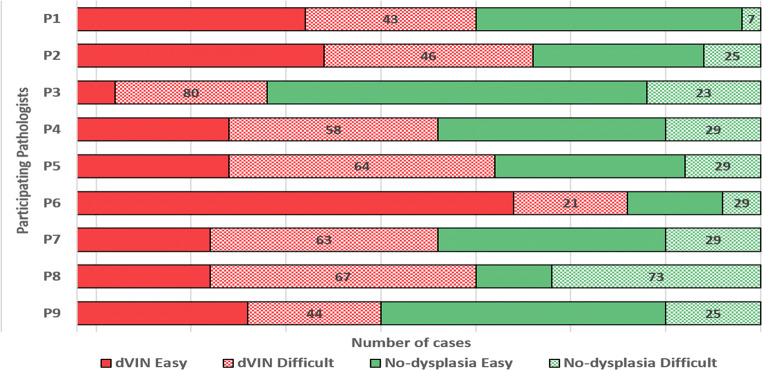
Bar chart depicting the proportions of slides diagnosed as dVIN or no-dysplasia per participant (P1–P9), along with the proportion of slides perceived as diagnostically difficult; the numbers depict the percentages of slides perceived as difficult

The participants diagnosed 28–81% (median = 58%) of the slides as dVIN, and perceived 21–80% (median = 58%) of these diagnoses to be difficult (Fig. 1). Nineteen to 72% (median = 42%) of the slides were diagnosed as no-dysplasia, of which 6–73% (median = 29%) were perceived as difficult.

#### Consensus diagnoses and diagnostic agreement for dVIN

**Fig. 2:**
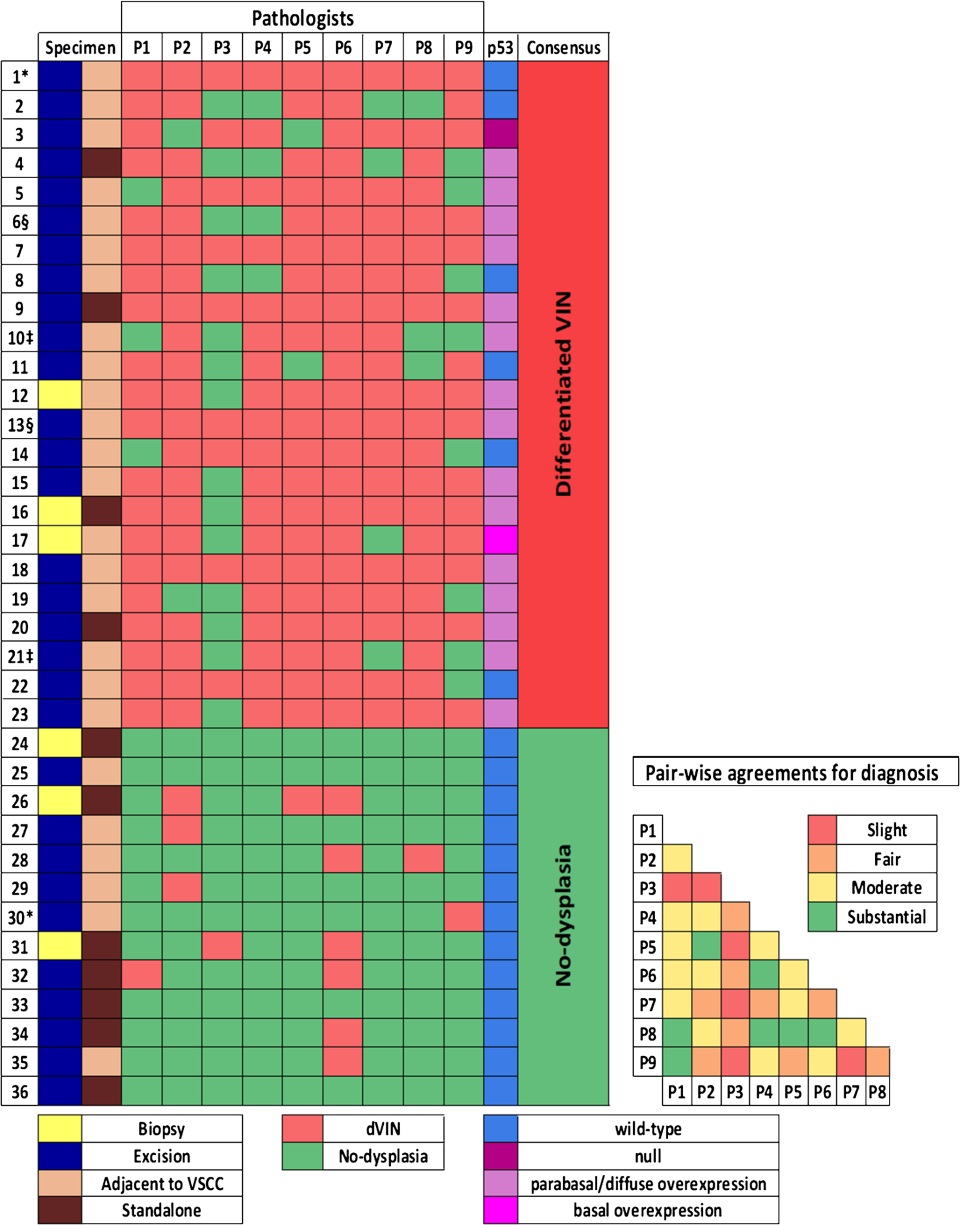
Left: Heat map depicting the types of specimens, diagnoses rendered by the participants (P1–P9), p53-IHC results, and the consensus diagnoses; *, ‡, § slides were from the same specimen. Right: Heat map depicting the levels of agreement between the participant pairs for the diagnosis; color-coding corresponds to the levels of agreement

The consensus diagnosis for 23 slides (64%) was dVIN (Fig. 2). For these slides, rates of diagnostic agreement ranged from 56 to 100% (median = 78%). Unanimous agreement (100%) was obtained for 5 slides.

The consensus diagnosis for 13 (36%) slides was no-dysplasia (Fig. 2). For these slides, rates of diagnostic agreement ranged from 67 to 100% (median = 89%). Unanimous agreement was obtained for 4 slides.

The overall agreement for the diagnosis of dVIN was moderate (*ĸ* = 0.42), and the pair-wise agreements ranged from slight (*ĸ* = 0.10) to substantial (*ĸ* = 0.73) (Table 1). Substantial agreement was obtained between 19%, and moderate agreement between 39% of the participant pairs (Fig. 2). Pair-wise *ĸ*-values with 95% CI are provided in Table S1. The diagnosis of dVIN was more frequently perceived to be difficult than the diagnosis of no-dysplasia (*p* = 0.02). For all slides (dVIN or no-dysplasia), diagnostic difficulty perceived by the participants correlated significantly with lower percentages of agreement (*p* = 0.001).

**Table 1:** Kappa values for the histological diagnoses and features of dVIN

	Overall agreement		Pair-wise agreements
	*ĸ*-values (95% CI)	Level of agreement	Range of *ĸ*-values
I. Histological diagnosis	0.42 (0.40–0.49)	Moderate	0.10–0.73
II. Histological features			
*A.* Nuclear atypia			
i. Atypia discernable under × 100 magnification	0.42 (0.33–0.46)	Moderate	0.06–0.70
ii. Angulated nuclei	0.32 (0.29–0.35)	Fair	0.01–0.65
iii. Macronucleoli	0.10 (0.04–0.13)	Slight	0.01–0.68
iv. Chromatin abnormality	0.32 (0.27–0.37)	Fair	0.08–0.67
v. Multinucleation	0.34 (0.32–0.41)	Fair	0.01–0.87
vi. Suprabasal mitoses	0.28 (0.22–0.31)	Fair	0.01–0.61
vii. Atypical mitoses	0.11 (0.04–0.14)	Slight	0.01–0.91
viii. Mitotic count >5/5 mm	0.45 (0.38–0.51)	Moderate	0.01–0.94
*B.* Disturbed maturation and architecture			
i. Individual cell keratinization	0.21 (0.17–0.24)	Fair	0.01–0.46
ii. Deep keratinization	0.19 (0.13–0.21)	Slight	0.01–0.48
iii. Deep squamous eddies	0.31 (0.23–0.42)	Fair	0.06–0.60
iv. Cobblestone appearance	0.22 (0.19–0.27)	Fair	0.10–0.61
v. Elongated and/or anastomosing rete ridges	0.30 (0.22–0.35)	Fair	0.04–0.69
vi. Altered cellular alignment	0.23 (0.18–0.29)	Fair	0.01–0.73
vii. Parakeratosis	0.57 (0.49–0.61)	Moderate	0.17–0.82

#### Correlation of the consensus diagnoses with p53-IHC patterns

Of the slides with a consensus diagnosis of dVIN, 17 (74%) showed p53-mutant patterns, which were basal to para-basal/diffuse overexpression in 15 slides, basal overexpression in 1 slide, and null-pattern in 1 slide. Six slides (26%) showed p53-wild-type pattern, i.e., scattered, heterogeneous, basal/para-basal expression (Fig. 2).

All slides with a consensus diagnosis of no-dysplasia showed p53-wild-type pattern, i.e., scattered, heterogeneous, basal/para-basal expression (Fig. 2). Concordance of the p53-IHC patterns with the consensus diagnoses was substantial (*ĸ* = 0.67; *p* < 0.001).

#### Agreements in the interpretation of histological features and ratings of their usefulness

Overall agreement was moderate in the interpretation of parakeratosis, mitotic count > 5/5 mm, and atypia discernable under × 100 magnification. Fair agreement was obtained for multinucleation, angulated nuclei, chromatin abnormality, suprabasal mitoses, deep squamous eddies, elongated and/or anastomosing rete ridges, altered cellular alignment, individual cell keratinization, and cobblestone appearance (Table 1).

Pair-wise agreements in the interpretation of the histological features ranged from slight (*ĸ* = 0.01) to near-perfect (*ĸ* = 0.94) (Table 1). The highest proportion of substantial/near-perfect agreement between participant pairs was obtained for parakeratosis (39%), and cobblestone appearance was rated most frequently (24%) as “very useful” for the diagnosis of dVIN (Table 2). Taking into consideration the levels of pair-wise agreements and the ratings of usefulness, the most helpful features were parakeratosis, cobblestone appearance, chromatin abnormality, angulated nuclei, atypia discernable under × 100, and altered cellular alignment.

**Table 2:** Histological features of dVIN, in descending order of the proportions of substantial/almost-perfect agreement, and ratings as “very useful” for diagnosis

Proportion of substantial/near-perfect agreement	Very useful for the diagnosis of dVIN
1. Parakeratosis (39%)	1. Cobblestone appearance (24%)
2. Mitotic count > 5/5 mm (19%)	2. Parakeratosis (19%)
3. Deep squamous eddies (14%)	3. Angulated nuclei (18%)
4. Multinucleation (11%)	4. Atypia discernable under × 100 (16%)
5. Chromatin abnormality (8%)	5. Chromatin abnormality (16%)
6. Atypical mitoses (8%)	6. Elongated and/or anastomosing rete ridges (13%)
7. Atypia discernable under × 100 (6%)	7. Altered cellular alignment (12%)
8. Angulated nuclei (6%)	8. Individual cell keratinization (12%)
9. Macronucleoli (6%)	9. Suprabasal mitoses (11%)
10. Cobblestone appearance (6%)	10. Deep keratinization (10%)
11. Altered cellular alignment (6%)	11. Macronucleoli (9%)
12. Elongated and/or anastomosing rete ridges (5%)	12. Multinucleation (3%)
13. Suprabasal mitoses (3%)	13. Mitotic count > 5/5 mm (3%)
14. Individual cell keratinization (0%)	14. Atypical mitoses (3%)
15. Deep keratinization (0%)	15. Deep squamous eddies (2%)

**Fig. 3:**
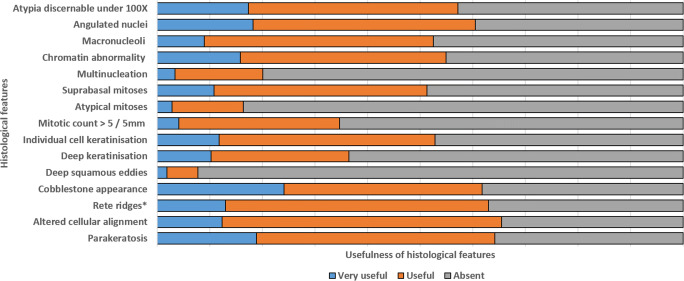
Bar charts representing the proportions of ratings of usefulness for each histological feature; *elongated and/or anastomosing rete ridges

**Fig. 4:**
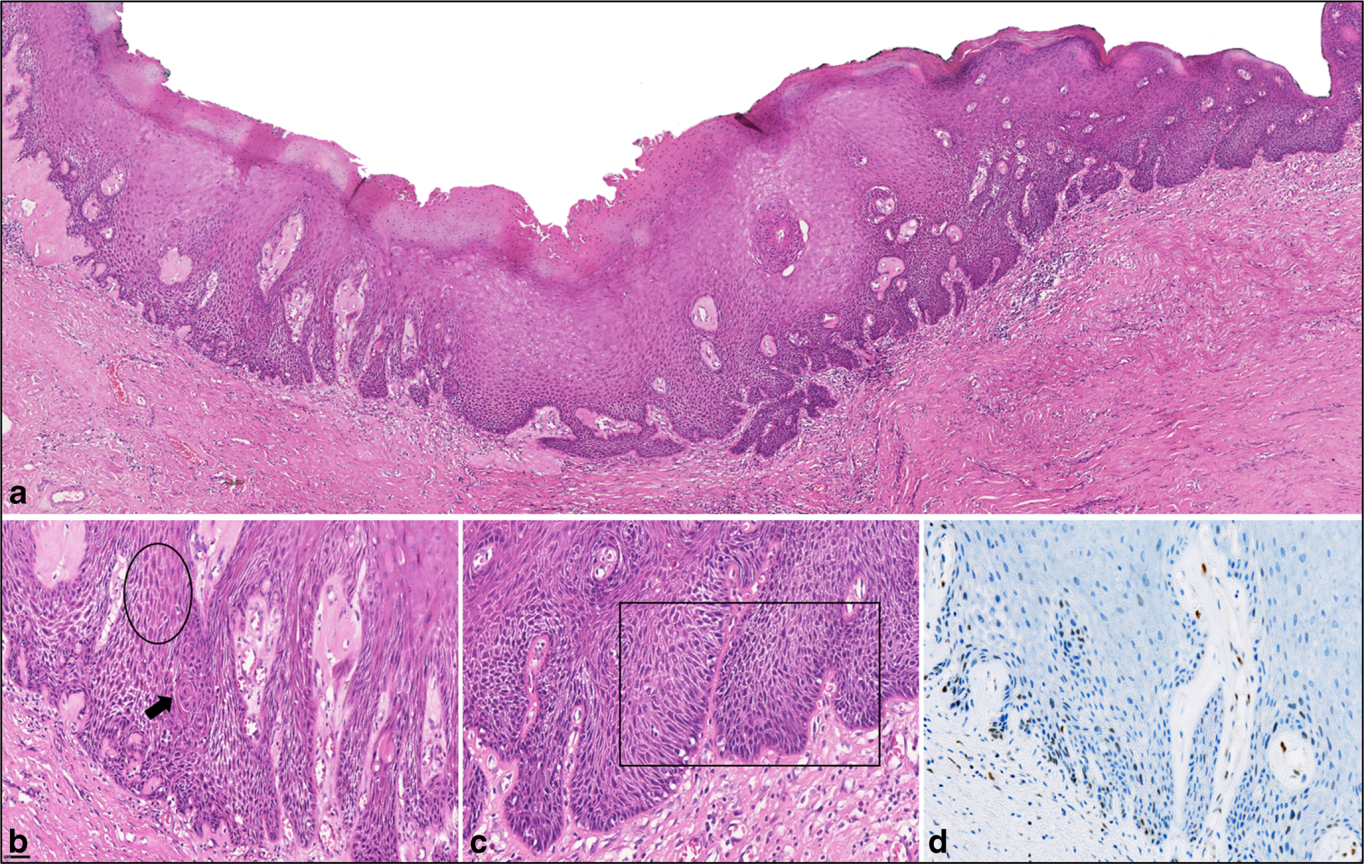
Low- (A) and high-magnification (B and C) images of one of the slides (HE-stain) that was diagnosed by all participants as dVIN, with corresponding p53-IHC (D). (A) Epithelial acanthosis, hyperkeratosis, parakeratosis, and an eosinophilic appearance can be appreciated under low magnification (original magnification × 5). (B) Cobblestone appearance (circled area), deep squamous eddies (arrow), elongated rete ridges, and (C) altered cellular alignment and angulated nuclei (squared area) were rated by the participants as “very useful” features for the diagnosis for this slide (original magnification × 200); (D) p53-IHC shows a wild-type pattern, i.e., scattered nuclear p53 staining of heterogeneous intensity in the basal and the para-basal layers

**Fig. 5:**
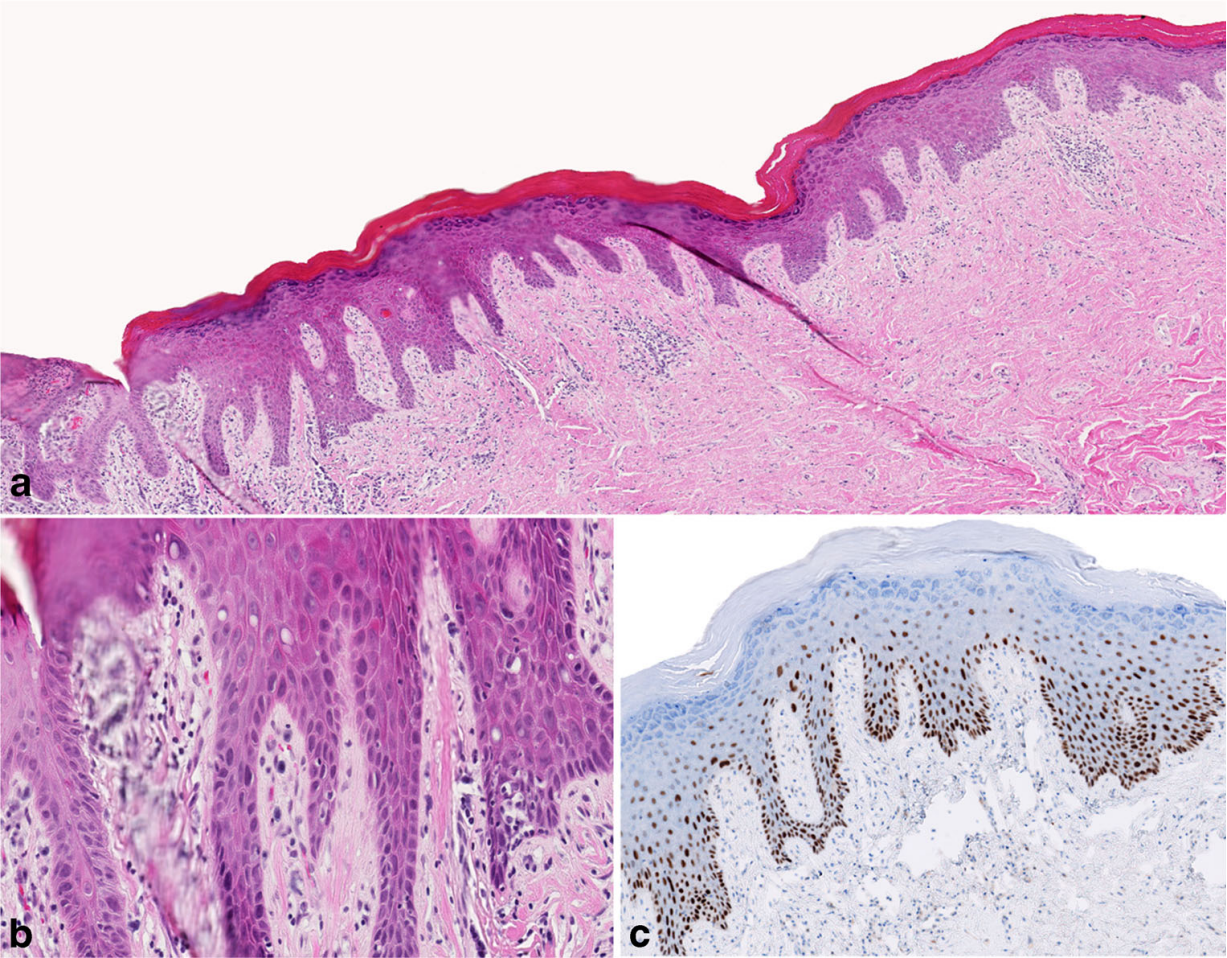
Low- (A) and high-magnification (B) images of one of the slides (HE-stain) that was diagnosed by 5 participants as dVIN and 4 participants as no-dysplasia, with corresponding p53-IHC (C). (A) Elongated rete ridges and hyperkeratosis are appreciable under low magnification (original magnification × 5). (B) Participants who diagnosed this slide as dVIN rated angulated nuclei, chromatin abnormality, cobblestone appearance, elongated rete ridges, and altered cellular alignment as “very useful” features for the diagnosis (original magnification × 300); (C) p53-IHC shows mutant pattern, i.e., diffuse, strong, nuclear p53 staining in the basal and para-basal layers

For each histological feature, the levels of pair-wise agreements are depicted in Figures S1 and S2, and the pair-wise *ĸ*-values with 95% CI are provided in Tables S2–S16. The ratings of usefulness are depicted in Fig. 3, and the histological features are demonstrated in Figs. 4 and 5.

## Discussion

To the best of our knowledge, this is the first bi-national, multi-institutional, ring-study to assess the inter-observer agreement in the histological assessment of dVIN. Agreement on the diagnosis between nine participating pathologists was moderate, while that between the participant pairs varied from slight to substantial. These results were similar to that of the only previous study on inter-observer agreement in dVIN [13], and indicate that the diagnostic agreement for dVIN remains suboptimal.

As histological diagnoses guide treatment decisions, variability in the diagnoses can result in treatment disparities [31]. Therefore, to improve the diagnostic reliability and to assure a similar standard of care, we suggest consensus evaluation of dVIN cases with a panel of pathologists experienced in vulvar neoplasia. Regular inter-disciplinary communication between gynecologists/dermatologists and pathologists can also enhance relevant knowledge and expertise.

An essential step to ensure a reliable histological diagnosis is to identify representative features which can be reproducibly interpreted by pathologists. We identified the most helpful features as parakeratosis, cobblestone appearance, chromatin abnormality, angulated nuclei, atypia discernable under × 100, and altered cellular alignment, based on the proportions of substantial/near-perfect agreement between the participant pairs, and the ratings of diagnostic usefulness. We observed that the participants recorded parakeratosis and cobblestone appearance as very useful for diagnosing dVIN, particularly where the nuclear atypia could not be discerned under × 100.

Previously, van den Einden et al. proposed that the presence of atypical mitoses in the basal layer, basal cellular atypia, dyskeratosis, prominent nucleoli, and elongated and anastomosing rete ridges were the most predictive features of dVIN [13]. In a subsequent survey among vulva pathology experts, only basal layer atypia was judged by consensus as an “essential” diagnostic feature [14]. However, neither of these studies assessed the agreement in the interpretation of these features. In our previous study, we obtained substantial agreement in the interpretation of macronucleoli, angulated nuclei, individual cell keratinization, deep keratinization, and deep squamous eddies, between pathologists at our center [15]. In the current study, however, similar level of agreement for these features was not observed. We speculate that our previous results may have been influenced by the similar standard of histological interpretation among participants who work in close collaboration at the same center.

In this study, we also correlated the histological consensus diagnoses with the immunohistochemical expression of p53, as this marker is commonly used to aid the diagnosis of dVIN. p53-mutant patterns have been reported to accurately reflect underlying *TP53* mutations, which characterize dVIN [19, 20, 32]. Substantial concordance of p53-IHC patterns with the histological consensus diagnoses was recorded, which confirms that routine use of this marker can improve the diagnostic accuracy for dVIN.

However, 6 (26%) of the slides in this study that were diagnosed as dVIN by consensus, showed wild-type p53-expression. This is in line with recent literature, which states that 17–42% cases of dVIN can show wild-type p53-expression [4], and implies that p53-IHC may not effectively inform the diagnosis in every case of dVIN. Furthermore, p53-IHC patterns in VSCC and the adjacent dVIN may not show perfect concordance [22]. A recent study reported that while dVIN adjacent to p53-wild-type VSCC always shows wild-type p53-expression, dVIN adjacent to p53-mutant VSCC can show wild-type p53-expression in 31.4% of cases [22]. In our study, all of the lesions judged as dVIN by consensus and showing wild-type p53-expression were present adjacent to VSCC. Similarly to the previous study [22], we observed that 67% (4/6) of these VSCCs showed wild-type p53-expression, while 33% (2/6) showed p53-mutant patterns (results not presented). This limitation of p53-IHC should be borne in mind particularly when using this marker to confirm the presence of dVIN in resection margins of VSCC. For dVINs that show wild-type p53-expression, the diagnosis defers to histological assessment, which, as our study indicates, may be fraught with variability. In view of this, we believe that ancillary biomarkers (immunohistochemical/molecular) need to be established to aid the diagnosis of the p53-wild-type subcategory of dVIN.

Through this study, we intended to estimate the diagnostic variability of dVIN in the real world. To ensure an accurate representation of this variability, (i) pathologists with varying levels of experience and from academic and non-academic centers were included, (ii) diagnostic criteria were not pre-determined to allow the participants to interpret the histology in light of their own experience, and (iii) assessments of outlier participants were not excluded.

Nevertheless, there are several limitations of this study. We used the majority (consensus) diagnosis of each slide to determine the diagnostic gold standard. It could be argued whether the consensus represents another diagnostic opinion rather than a standard of truth. dVIN is known to originate in a background of chronic dermatoses, and there is no clear, universally accepted threshold for identifying atypia/dysplasia. This threshold is often influenced by the pathologists’ training and/or practice experience. Unless a reliable IHC marker is established, every method to ascertain a gold-standard diagnosis will have some bias.

There is also little consensus on the ideal method for measuring observer agreement in pathology diagnosis. It has been suggested that both percentages of agreement and *ĸ*-statistics do not take into account the prevalence of a particular diagnosis in a set of cases, or completely rule out concordances due to chance [33, 34]. Validity of the cut-offs that are used to interpret levels of agreement from *ĸ*-values has also been challenged [30, 35].

It could also be argued whether our study over-estimated the diagnostic variability. Unlike in routine practice, participants diagnosed the slides without clinical information, serial sections, or IHC. The selection contained a higher proportion of dVIN than no-dysplasia slides, which may not reflect routine practice. We lacked statistical power to evaluate the influence of level of experience or practice setting on the diagnostic variability. Furthermore, the inter-observer agreement in the interpretation of p53-IHC was not assessed. To gain further insights on these contexts, we have set up a larger study among geographically disparate group of pathologists, which includes the assessment of p53-IHC.

In conclusion, the suboptimal level of diagnostic agreement for dVIN observed in this study affirms the difficulty of the diagnosis. We identified parakeratosis, cobblestone appearance, chromatin abnormality, angulated nuclei, atypia discernable under × 100, and altered cellular alignment as helpful diagnostic features of dVIN. For cases with a histological suspicion of dVIN, we suggest consensus-based pathological evaluation to improve diagnostic reliability.

## Supplementary Information

ESM 1 (DOCX 16.3 kb)

ESM 2 (DOCX 22 kb)

ESM 3 (DOCX 42 kb)

Figure S1: Heat maps depicting the levels of agreement between the participant pairs for the features of nuclear atypia; color-coding corresponds to the levels of agreement (PNG 376 kb)

High Resolution Image (TIF 14061 kb)

Figure S2: Heat maps depicting the levels of agreement between the participant pairs for the features of disturbed maturation / architecture; color-coding corresponds to the levels of agreement; *elongated and / or anastomosing rete ridges (PNG 499 kb)

High Resolution Image (TIF 16.3 mb)

## Data Availability

Whole slide images of the cases included in this study are available for sharing with physicians and researchers for educational and research purposes. Upon reasonable request, images will be shared in a secure manner, obeying our hospital guidelines. Requests can be directed to the corresponding author.
