# Supplementary material for: Histological interpretation of differentiated vulvar intraepithelial neoplasia (dVIN) remains challenging—observations from a bi-national ring-study
Source: Virchows Arch. 2021 Mar 8;479(2):305–15. doi: 10.1007/s00428-021-03070-0 (PMC8364542; doi:10.1007/s00428-021-03070-0)
Supplement: Supplementary file 1 — (DOCX 16.3 kb) [file 428_2021_3070_MOESM1_ESM.docx]

# **Supplementary document 1**

# **Immunohistochemistry (IHC) protocol – p16 and p53**

Automated IHC was performed using the Ventana Benchmark ULTRA (Ventana Medical Systems Inc.). Sequential sections of 4 µm thickness were prepared from the formalin fixed paraffin embedded tissues and stained with p16 and p53 using Optiview universal DAB detection Kit (#760-700, Ventana).

In brief, following de-paraffinization and heat-induced antigen retrieval with CC1 (#950-500, Ventana) for 64 minutes, the tissue samples were incubated with the antibody of interest for 32 minutes at 37˚C. Incubation was followed by hematoxylin II counter stain for 8 minutes and then a blue coloring reagent for 8 minutes according to the manufactures instructions (Ventana). Tonsil tissues were used as positive controls for every slide. Additional relevant information on the antibodies are tabulated below.

| **Antibody** | **Type** | **Dilution** | **Company** | **Clone** |
| --- | --- | --- | --- | --- |
| p16 | Anti-mouse | 1.0 µg/ml | Ventana | E6H4 |
| p53 | Anti-mouse | 2.5 µg/ml | Ventana | Bp53 -11 |
