# Supplementary material for: Histological interpretation of differentiated vulvar intraepithelial neoplasia (dVIN) remains challenging—observations from a bi-national ring-study
Source: Virchows Arch. 2021 Mar 8;479(2):305–15. doi: 10.1007/s00428-021-03070-0 (PMC8364542; doi:10.1007/s00428-021-03070-0)
Supplement: Supplementary file 2 — (DOCX 22 kb) [file 428_2021_3070_MOESM2_ESM.docx]

# **Supplementary document 2**

## **Assessment form**

|  | **Cases** | | | | | | | | | | | | | | | | | | | | | | | | | | | | | | | | | | | | | | |
| --- | --- | --- | --- | --- | --- | --- | --- | --- | --- | --- | --- | --- | --- | --- | --- | --- | --- | --- | --- | --- | --- | --- | --- | --- | --- | --- | --- | --- | --- | --- | --- | --- | --- | --- | --- | --- | --- | --- | --- |
|  | **1** | **2** | | **3** | | **4** | | **5** | | **6** | | **7** | | **8** | | **9** | | **10** | | **11** | | **12** | | **13** | | **14** | | **15** | | **16** | | **17** | | **18** | | **19** | | **20** | |
| **Diagnosis** [Categories: 1 - dVIN, 2 - No dysplasia] |  |  | |  | |  | |  | |  | |  | |  | |  | |  | |  | |  | |  | |  | |  | |  | |  | |  | |  | |  | |
| **Histological features** [Scoring guide: 0 - Not present; 1 – Present, Useful; 3 – Present, Very useful] | | | | | | | | | | | | | | | | | | | | | | | | | | | | | | | | | | | | | | | |
| ***Nuclear atypia*** | | | | | | | | | | | | | | | | | | | | | | | | | | | | | | | | | | | | | | | |
| 1. Atypia discernable under 100X magnification |  | |  | |  | |  | |  | |  | |  | |  | |  | |  | |  | |  | |  | |  | |  | |  | |  | |  | |  | |  |
| 1. Angulated nuclei |  | |  | |  | |  | |  | |  | |  | |  | |  | |  | |  | |  | |  | |  | |  | |  | |  | |  | |  | |  |
| 1. Macronucleoli |  | |  | |  | |  | |  | |  | |  | |  | |  | |  | |  | |  | |  | |  | |  | |  | |  | |  | |  | |  |
| 1. Chromatin abnormality |  | |  | |  | |  | |  | |  | |  | |  | |  | |  | |  | |  | |  | |  | |  | |  | |  | |  | |  | |  |
| 1. Multinucleation |  | |  | |  | |  | |  | |  | |  | |  | |  | |  | |  | |  | |  | |  | |  | |  | |  | |  | |  | |  |
| 1. Suprabasal mitoses |  | |  | |  | |  | |  | |  | |  | |  | |  | |  | |  | |  | |  | |  | |  | |  | |  | |  | |  | |  |
| 1. Atypical mitoses |  | |  | |  | |  | |  | |  | |  | |  | |  | |  | |  | |  | |  | |  | |  | |  | |  | |  | |  | |  |
| 1. Mitotic count > 5 / 5mm |  | |  | |  | |  | |  | |  | |  | |  | |  | |  | |  | |  | |  | |  | |  | |  | |  | |  | |  | |  |
| ***Features of disturbed maturation and architecture*** | | | | | | | | | | | | | | | | | | | | | | | | | | | | | | | | | | | | | | | |
| 1. Individual cell keratinization |  | |  | |  | |  | |  | |  | |  | |  | |  | |  | |  | |  | |  | |  | |  | |  | |  | |  | |  | |  |
| 1. Deep keratinization |  | |  | |  | |  | |  | |  | |  | |  | |  | |  | |  | |  | |  | |  | |  | |  | |  | |  | |  | |  |
| 1. Deep squamous eddies |  | |  | |  | |  | |  | |  | |  | |  | |  | |  | |  | |  | |  | |  | |  | |  | |  | |  | |  | |  |
| 1. Cobblestone appearance |  | |  | |  | |  | |  | |  | |  | |  | |  | |  | |  | |  | |  | |  | |  | |  | |  | |  | |  | |  |
| 1. Elongated and / or anastomosing rete ridges |  | |  | |  | |  | |  | |  | |  | |  | |  | |  | |  | |  | |  | |  | |  | |  | |  | |  | |  | |  |
| 1. Altered cellular alignment |  | |  | |  | |  | |  | |  | |  | |  | |  | |  | |  | |  | |  | |  | |  | |  | |  | |  | |  | |  |
| 1. Parakeratosis |  | |  | |  | |  | |  | |  | |  | |  | |  | |  | |  | |  | |  | |  | |  | |  | |  | |  | |  | |  |
| **Difficulty in diagnosis** [1 - easy, 2 - difficult] |  | |  | |  | |  | |  | |  | |  | |  | |  | |  | |  | |  | |  | |  | |  | |  | |  | |  | |  | |  |

|  | **Cases** | | | | | | | | | | | | | | | | | | | |
| --- | --- | --- | --- | --- | --- | --- | --- | --- | --- | --- | --- | --- | --- | --- | --- | --- | --- | --- | --- | --- |
|  | **21** | **22** | **23** | **24** | **25** | **26** | **27** | **28** | **29** | **30** | **31** | **32** | **33** | **34** | **35** | **36** | **37** | **38** | **39** | **40** |
| **Diagnosis** [Categories: 1 - dVIN, 2 - No dysplasia] |  |  |  |  |  |  |  |  |  |  |  |  |  |  |  |  |  |  |  |  |
| **Histological features** [Scoring guide: 0 - Not present; 1 – Present, Useful; 3 – Present, Very useful] | | | | | | | | | | | | | | | | | | | | |
| ***Nuclear atypia*** | | | | | | | | | | | | | | | | | | | | |
| 1. Atypia discernable under 100X magnification |  |  |  |  |  |  |  |  |  |  |  |  |  |  |  |  |  |  |  |  |
| 1. Angulated nuclei |  |  |  |  |  |  |  |  |  |  |  |  |  |  |  |  |  |  |  |  |
| 1. Macronucleoli |  |  |  |  |  |  |  |  |  |  |  |  |  |  |  |  |  |  |  |  |
| 1. Chromatin abnormality |  |  |  |  |  |  |  |  |  |  |  |  |  |  |  |  |  |  |  |  |
| 1. Multinucleation |  |  |  |  |  |  |  |  |  |  |  |  |  |  |  |  |  |  |  |  |
| 1. Suprabasal mitoses |  |  |  |  |  |  |  |  |  |  |  |  |  |  |  |  |  |  |  |  |
| 1. Atypical mitoses |  |  |  |  |  |  |  |  |  |  |  |  |  |  |  |  |  |  |  |  |
| 1. Mitotic count > 5 / 5mm |  |  |  |  |  |  |  |  |  |  |  |  |  |  |  |  |  |  |  |  |
| ***Features of disturbed maturation and architecture*** | | | | | | | | | | | | | | | | | | | | |
| 1. Individual cell keratinization |  |  |  |  |  |  |  |  |  |  |  |  |  |  |  |  |  |  |  |  |
| 1. Deep keratinization |  |  |  |  |  |  |  |  |  |  |  |  |  |  |  |  |  |  |  |  |
| 1. Deep squamous eddies |  |  |  |  |  |  |  |  |  |  |  |  |  |  |  |  |  |  |  |  |
| 1. Cobblestone appearance |  |  |  |  |  |  |  |  |  |  |  |  |  |  |  |  |  |  |  |  |
| 1. Elongated and / or anastomosing rete ridges |  |  |  |  |  |  |  |  |  |  |  |  |  |  |  |  |  |  |  |  |
| 1. Altered cellular alignment |  |  |  |  |  |  |  |  |  |  |  |  |  |  |  |  |  |  |  |  |
| 1. Parakeratosis |  |  |  |  |  |  |  |  |  |  |  |  |  |  |  |  |  |  |  |  |
| **Difficulty in diagnosis** [1 - easy, 2 - difficult] |  |  |  |  |  |  |  |  |  |  |  |  |  |  |  |  |  |  |  |  |

## **Participant information**

| **Name of participant** |  |
| --- | --- |
| **Country of practice** | 1. The Netherlands 2. Belgium 3. Both |
| **Years in practice**  **(excluding years in training)** | 1. < 5 years 2. 5 – 10 years 3. 10 – 15 years 4. > 15 years |
| **Type of practice** | 1. Academic 2. Non-academic 3. Both |
| **Degree(s)** |  |
| **Current affiliation(s)** |  |
