# Supplementary material for: Histological interpretation of differentiated vulvar intraepithelial neoplasia (dVIN) remains challenging—observations from a bi-national ring-study
Source: Virchows Arch. 2021 Mar 8;479(2):305–15. doi: 10.1007/s00428-021-03070-0 (PMC8364542; doi:10.1007/s00428-021-03070-0)
Supplement: Supplementary file 3 — (DOCX 42 kb) [file 428_2021_3070_MOESM3_ESM.docx]

# **Supplementary document 3**

## **Table S1: Pair-wise Kappa (ĸ)-values with 95% confidence intervals (CI) for the histological diagnosis of dVIN**

| **P1** |  |  |  |  |  |  |  |  |
| --- | --- | --- | --- | --- | --- | --- | --- | --- |
| **P2** | 0.47 (0.14-0.79) |  |  |  |  |  |  |  |
| **P3** | 0.14 (0.02-0.36) | 0.10 (0.001-0.32) |  |  |  |  |  |  |
| **P4** | 0.55 (0.22-0.87) | 0.48 (0.15-0.80) | 0.36 (0.03-0.69) |  |  |  |  |  |
| **P5** | 0.60 (0.26-0.92) | 0.72 (0.43-1.08) | 0.11 (0.02-0.42) | 0.60 (0.27-0.93) |  |  |  |  |
| **P6** | 0.55 (0.22-0.87) | 0.48 (0.15-0.80) | 0.36 (0.03-0.69) | 0.73 (0.45-1.10) | 0.60 (0.27-0.93) |  |  |  |
| **P7** | 0.48 (0.14-0.80) | 0.36 (0.02-0.68) | 0.16 (0.03-0.26) | 0.38 (0.04-0.70) | 0.53 (0.20-0.85) | 0.38 (0.04-0.70) |  |  |
| **P8** | 0.66 (0.33-0.98) | 0.47 (0.14-0.79) | 0.26 (0.01-0.59) | 0.66 (0.33-0.98) | 0.71 (0.38-1.03) | 0.66 (0.33-0.98) | 0.48 (0.14-0.80) |  |
| **P9** | 0.61 (0.28-0.93) | 0.33 (0.01-0.65) | 0.16 (0.01-0.48) | 0.51 (0.17-0.82) | 0.33 (0.01-0.65) | 0.50 (0.17-0.82) | 0.10 (0.20-0.43) | 0.39 (0.06-0.71) |
|  | **P1** | **P2** | **P3** | **P4** | **P5** | **P6** | **P7** | **P8** |

**Tables S2-S16: Pair-wise ĸ-values with 95% CIs for the interpretation of the individual histological features**

## **Table S2: Atypia discernible under 100X**

| **P1** |  |  |  |  |  |  |  |  |
| --- | --- | --- | --- | --- | --- | --- | --- | --- |
| **P2** | 0.25 (0.07-0.57) |  |  |  |  |  |  |  |
| **P3** | 0.33 (0.01-0.65) | 0.06 (0.01-0.27) |  |  |  |  |  |  |
| **P4** | 0.44 (0.11-0.76) | 0.38 (0.05-0.70) | 0.43 (0.10-0.75) |  |  |  |  |  |
| **P5** | 0.55 (0.22-0.88) | 0.49 (0.16-0.81) | 0.09 (0.04-0.41) | 0.56 (0.22-0.88) |  |  |  |  |
| **P6** | 0.13 (0.01-0.45) | 0.11 (0.01-0.43) | 0.10 (0.02-0.22) | 0.17 (0.05-0.49) | 0.17 (0.05-0.49) |  |  |  |
| **P7** | 0.59 (0.25-0.91) | 0.50 (0.17-0.82) | 0.17 (0.13-0.49) | 0.48 (0.15-0.80) | 0.48 (0.15-0.80) | 0.36 (0.03-0.68) |  |  |
| **P8** | 0.60 (0.27-0.93) | 0.42 (0.08-0.74) | 0.27 (0.05-0.59) | 0.70 (0.39-1.04) | 0.50 (0.16-0.82) | 0.31 (0.01-0.63) | 0.52 (0.19-0.84) |  |
| **P9** | 0.44 (0.11-0.77) | 0.38 (0.05-0.71) | 0.30 (0.02-0.62) | 0.55 (0.22-0.88) | 0.55 (0.22-0.88) | 0.08 ((-0.24)-0.40) | 0.49 (0.16-0.81) | 0.61 (0.28-0.93) |
|  | **P1** | **P2** | **P3** | **P4** | **P5** | **P6** | **P7** | **P8** |

## **Table S3: Angulated nuclei**

| **P1** |  |  |  |  |  |  |  |  |
| --- | --- | --- | --- | --- | --- | --- | --- | --- |
| **P2** | 0.55 (0.22-0.87) |  |  |  |  |  |  |  |
| **P3** | 0.20 (0.12-0.52) | 0.27 (0.05-0.59) |  |  |  |  |  |  |
| **P4** | 0.43 (0.10-0.75) | 0.50 (0.16-0.82) | 0.55 (0.22-0.87) |  |  |  |  |  |
| **P5** | 0.33 (0.01-0.65) | 0.39 (0.06-0.71) | 0.31 (0.01-0.64) | 0.54 (0.21-0.86) |  |  |  |  |
| **P6** | 0.03 (0.01-0.36) | 0.24 (0.06-0.68) | 0.28 (0.20-0.74) | 0.28 (0.21-0.46) | 0.34 (0.26-0.71) |  |  |  |
| **P7** | 0.53 (0.20-0.85) | 0.38 (0.04-0.70) | 0.38 (0.05-0.70) | 0.49 (0.16-0.81) | 0.27 (0.05-0.59) | 0.01 (0.001-0.43) |  |  |
| **P8** | 0.65 (0.36-1.02) | 0.53 (0.20-0.85) | 0.50 (0.17-0.82) | 0.65 (0.28-0.93) | 0.39 (0.06-0.71) | 0.13 (0.05-0.62) | 0.40 (0.27-0.72) |  |
| **P9** | 0.45 (0.12-0.77) | 0.42 (0.08-0.74) | 0.28 (0.04-0.60) | 0.39 (0.06-0.71) | 0.17 (0.16-0.49) | 0.13 (0.05-0.62) | 0.28 (0.14-0.60) | 0.54 (0.21-0.86) |
|  | **P1** | **P2** | **P3** | **P4** | **P5** | **P6** | **P7** | **P8** |

## **Table S4: Macronucleoli**

| **P1** |  |  |  |  |  |  |  |  |
| --- | --- | --- | --- | --- | --- | --- | --- | --- |
| **P2** | 0.55 (0.22-0.87) |  |  |  |  |  |  |  |
| **P3** | 0.29 (0.06-0.34) | 0.11 (0.4-0.21) |  |  |  |  |  |  |
| **P4** | 0.05 (0.01-0.37) | 0.20 (0.12-0.53) | 0.03 ((-0.29)-0.35) |  |  |  |  |  |
| **P5** | 0.05 (0.01-0.38) | 0.35 (0.02-0.67) | 0.24 (0.01-0.56) | 0.31 (0.01-0.63) |  |  |  |  |
| **P6** | 0.01 ((-0.29)-0.36) | 0.13 (0.05-0.45) | 0.68 (0.46-0.70) | 0.42 (0.24-0.89) | 0.56 (0.24-0.67) |  |  |  |
| **P7** | 0.24 (0.08-0.56) | 0.09 (0.04-0.41) | 0.61 (0.47-0.68) | 0.37 (0.19-0.76) | 0.29 (0.14-0.49) | 0.11 (0.01-0.43) |  |  |
| **P8** | 0.55 (0.22-0.87) | 0.49 (0.16-0.81) | 0.33 (0.06-0.40) | 0.01 ((-0.02)-0.32) | 0.11 (0.08-0.43) | 0.07 (0.05-0.40) | 0.11 (0.02-0.43) |  |
| **P9** | 0.26 (0.06-0.59) | 0.50 (0.16-0.82) | 0.01 ((-0.3)-0.30)) | 0.16 (0.08-0.48) | 0.18 (0.14-0.50) | 0.09 ((-0.24)-0.41) | 0.05 (0.01-0.37) | 0.20 (0.12-0.52) |
|  | **P1** | **P2** | **P3** | **P4** | **P5** | **P6** | **P7** | **P8** |

## **Table S5: Chromatin abnormality**

| **P1** |  |  |  |  |  |  |  |  |
| --- | --- | --- | --- | --- | --- | --- | --- | --- |
| **P2** | 0.48 (0.14-0.80) |  |  |  |  |  |  |  |
| **P3** | 0.08 (0.04-0.25) | 0.08 (0.04-0.25) |  |  |  |  |  |  |
| **P4** | 0.36 (0.03-0.69) | 0.48 (0.15-0.80) | 0.25 (0.07-0.57) |  |  |  |  |  |
| **P5** | 0.27 (0.05-0.59) | 0.27 (0.05-0.59) | 0.17 (0.01-0.49) | 0.67 (0.33-0.98) |  |  |  |  |
| **P6** | 0.31 (0.01-0.63) | 0.17 (0.01-0.49) | 0.17 (0.05-0.15) | 0.47 (0.14-0.79) | 0.15 (0.01-0.47) |  |  |  |
| **P7** | 0.35 (0.01-0.67) | 0.21 (0.11-0.54) | 0.08 (0.04-0.25) | 0.48 (0.15-0.80) | 0.38 (0.05-0.71) | 0.17 (0.01-0.49) |  |  |
| **P8** | 0.42 (0.08-0.74) | 0.42 (0.08-0.74) | 0.08 (0.02-0.40) | 0.61 (0.28-0.93) | 0.38 (0.05-0.71) | 0.40 (0.07-0.72) | 0.30 (0.02-0.62) |  |
| **P9** | 0.43 (0.10-0.75) | 0.43 (0.10-0.75) | 0.30 (0.02-0.62) | 0.61 (0.28-0.93) | 0.26 (0.06-0.59) | 0.30 (0.02-0.62) | 0.31 (0.01-0.64) | 0.56 (0.22-0.88) |
|  | **P1** | **P2** | **P3** | **P4** | **P5** | **P6** | **P7** | **P8** |

## **Table S6: Multinucleation**

| **P1** |  |  |  |  |  |  |  |  |
| --- | --- | --- | --- | --- | --- | --- | --- | --- |
| **P2** | 0.29 (0.03-0.61) |  |  |  |  |  |  |  |
| **P3** | 0.46 (0.13-0.79) | 0.25 (0.07-0.57) |  |  |  |  |  |  |
| **P4** | 0.46 (0.13-0.79) | 0.25 (0.07-0.57) | 0.72 (0.39-1.04) |  |  |  |  |  |
| **P5** | 0.54 (0.20-0.86) | 0.54 (0.20-0.86) | 0.53 (0.19-0.85) | 0.53 (0.19-0.85) |  |  |  |  |
| **P6** | 0.30 (0.02-0.62) | 0.30 (0.02-0.62) | 0.53 (0.19-0.85) | 0.87 (0.51-1.16) | 0.64 (0.30-0.96) |  |  |  |
| **P7** | 0.45 (0.11-0.77) | 0.31 (0.01-0.63) | 0.31 (0.01-0.63) | 0.31 (0.01-0.63) | 0.20 (0.13-0.52) | 0.20 (0.13-0.52) |  |  |
| **P8** | 0.46 (0.13-0.78) | 0.61 (0.28-0.94) | 0.47 (0.14-0.79) | 0.47 (0.14-0.79) | 0.34 (0.01-0.66) | 0.34 (0.01-0.66) | 0.55 (0.22-0.87) |  |
| **P9** | 0.17 (0.01-0.49) | 0.45 (0.11-0.77) | 0.01 (0.005-0.32) | 0.01 (0.006-0.32) | 0.20 (0.13-0.52) | 0.04 (0.01-0.36) | 0.28 (0.04-0.60) | 0.43 (0.09-0.75) |
|  | **P1** | **P2** | **P3** | **P4** | **P5** | **P6** | **P7** | **P8** |

## **Table S7: Suprabasal mitoses**

| **P1** |  |  |  |  |  |  |  |  |
| --- | --- | --- | --- | --- | --- | --- | --- | --- |
| **P2** | 0.50 (0.17-0.82) |  |  |  |  |  |  |  |
| **P3** | 0.01 ((-0.3)-0.31) | 0.19 (0.05-0.13) |  |  |  |  |  |  |
| **P4** | 0.27 (0.05-0.59) | 0.17 (0.01-0.49) | 0.20 ((-0.1)-0.52) |  |  |  |  |  |
| **P5** | 0.28 (0.04-0.60) | 0.38 (0.05-0.70) | 0.03 ((-0.2)-0.35) | 0.52 (0.19-0.84) |  |  |  |  |
| **P6** | 0.21 (0.11-0.54) | 0.26 (0.06-0.58) | 0.29 ((-0.6)-0.03) | 0.05 (0.02-0.37) | 0.25 (0.07-0.57) |  |  |  |
| **P7** | 0.52 (0.19-0.84) | 0.48 (0.14-0.80) | 0.10 (0.04-0.22) | 0.28 (0.04-0.60) | 0.38 (0.05-0.71) | 0.45 (0.11-0.77) |  |  |
| **P8** | 0.54 (0.21-0.86) | 0.52 (0.19-0.84) | 0.08 (0.02-0.40) | 0.49 (0.16-0.81) | 0.50 (0.17-0.82) | 0.38 (0.04-0.70) | 0.61 (0.32-0.97) |  |
| **P9** | 0.05 (0.02-0.37) | 0.13 (0.01-0.46) | 0.08 (0.02-0.41) | 0.30 (0.02-0.62) | 0.33 (0.00-0.65) | 0.11 (0.02-0.43) | 0.26 (0.06-0.59) | 0.28 (0.04-0.60) |
|  | **P1** | **P2** | **P3** | **P4** | **P5** | **P6** | **P7** | **P8** |

## **Table S8: Atypical mitoses**

| **P1** |  |  |  |  |  |  |  |  |
| --- | --- | --- | --- | --- | --- | --- | --- | --- |
| **P2** | 0.03 ((-0.29)-0.36) |  |  |  |  |  |  |  |
| **P3** | 0.14 ((-0.46)-0.18) | 0.03 ((-0.35)-0.29) |  |  |  |  |  |  |
| **P4** | 0.07 ((-0.25)-0.39) | 0.65 (0.33-0.97) | 0.01 ((-0.34)-0.31) |  |  |  |  |  |
| **P5** | 0.20 ((-0.12)-0.53) | 0.36 (0.03-0.68) | 0.04 ((-0.37)-0.28) | 0.47 (0.14-0.79) |  |  |  |  |
| **P6** | 0.07 ((-0.25)-0.39) | 0.65 (0.325-0.97) | 0.01 ((-0.34)-0.31) | 0.91 (0.67-1.33) | 0.47 (0.14-0.79) |  |  |  |
| **P7** | 0.39 (0.07-0.72) | 0.06 (0.04-0.26) | 0.20 ((-0.52)-0.26) | 0.03 ((-0.35)-0.29) | 0.07 ((-0.25)-0.40) | 0.03 ((-0.35)-0.29) |  |  |
| **P8** | 0.07 (0.03-0.39) | 0.01 ((-0.32)-0.32) | 0.16 ((-0.48)-0.20) | 0.03 ((-0.29)-0.36) | 0.03 ((-0.35)-0.29) | 0.03 ((-0.29)-0.36) | 0.48 (0.15-0.80) |  |
| **P9** | 0.30 (0.03-0.63) | 0.29 ((-0.61)-0.40) | 0.24 ((-0.56)-0.35) | 0.26 ((-0.58)-0.36) | 0.31 ((-0.63)-0.02) | 0.26 ((-0.58)-0.36) | 0.40 (0.07-0.72) | 0.13 (-0.20-0.45) |
|  | **P1** | **P2** | **P3** | **P4** | **P5** | **P6** | **P7** | **P8** |

## **Table S9: Mitotic count > 5 / 5mm**

| **P1** |  |  |  |  |  |  |  |  |
| --- | --- | --- | --- | --- | --- | --- | --- | --- |
| **P2** | 0.61 (0.28-0.94) |  |  |  |  |  |  |  |
| **P3** | 0.07 (0.02-0.39) | 0.14 (0.04-0.18) |  |  |  |  |  |  |
| **P4** | 0.94 (0.52-1.17) | 0.77 (0.44-1.09) | 0.07 (0.02-0.39) |  |  |  |  |  |
| **P5** | 0.70 (0.37-1.03) | 0.92 (0.59-1.24) | 0.16 (0.04-0.16) | 0.85 (0.52-1.17) |  |  |  |  |
| **P6** | 0.58 (0.25-0.91) | 0.50 (0.17-0.82) | 0.01 (-0.3-0.32) | 0.58 (0.25-0.91) | 0.58 (0.25-0.91) |  |  |  |
| **P7** | 0.52 (0.19-0.84) | 0.45 (0.12-0.77) | 0.19 (0.05-0.14) | 0.52 (0.19-0.84) | 0.52 (0.19-0.84) | 0.53 (0.20-0.85) |  |  |
| **P8** | 0.52 (0.19-0.84) | 0.45 (0.12-0.77) | 0.19 (0.05-0.14) | 0.52 (0.19-0.84) | 0.52 (0.19-0.84) | 0.53 (0.20-0.85) | 0.78 (0.45-1.10) |  |
| **P9** | 0.21 (0.11-0.53) | 0.15 (0.01-0.47) | 0.38 (0.07-0.45) | 0.21 (0.11-0.53) | 0.21 (0.11-0.53) | 0.33 (0.01-0.65) | 0.44 (0.11-0.76) | 0.33 (0.01-0.65) |
|  | **P1** | **P2** | **P3** | **P4** | **P5** | **P6** | **P7** | **P8** |

## **Table S10: Individual cell keratinization**

| **P1** |  |  |  |  |  |  |  |  |
| --- | --- | --- | --- | --- | --- | --- | --- | --- |
| **P2** | 0.23 (0.09-0.55) |  |  |  |  |  |  |  |
| **P3** | 0.20 (0.12-0.52) | 0.17 (0.01-0.49) |  |  |  |  |  |  |
| **P4** | 0.06 ((-0.3)-0.27) | 0.20 (0.12-0.52) | 0.11 (0.02-0.43) |  |  |  |  |  |
| **P5** | 0.30 (0.02-0.62) | 0.27 (0.05-0.59) | 0.44 (0.11-0.77) | 0.25 (0.07-0.57) |  |  |  |  |
| **P6** | 0.24 (0.08-0.56) | 0.03 ((-0.2)-0.35) | 0.04 ((-0.2)-0.36) | 0.26 (0.05-0.06) | 0.13 ((-0.2)-0.45) |  |  |  |
| **P7** | 0.13 (0.02-0.45) | 0.28 (0.04-0.60) | 0.13 (0.01-0.46) | 0.01 ((-0.3)-0.32) | 0.23 (0.09-0.55) | 0.29 (0.03-0.61) |  |  |
| **P8** | 0.38 (0.05-0.71) | 0.22 (0.10-0.54) | 0.38 (0.05-0.70) | 0.38 (0.04-0.70) | 0.46 (0.16-0.82) | 0.08 ((-0.4)-0.24) | 0.21 (0.11-0.53) |  |
| **P9** | 0.06 (0.03-0.26) | 0.06 (0.02-0.39) | 0.33 (0.01-0.65) | 0.41 (0.08-0.73) | 0.23 (0.09-0.55) | 0.35 (0.06-0.40) | 0.01 ((-0.3)-0.32) | 0.46 (0.14-0.80) |
|  | **P1** | **P2** | **P3** | **P4** | **P5** | **P6** | **P7** | **P8** |

## **Table S11: Deep keratinization**

| **P1** |  |  |  |  |  |  |  |  |
| --- | --- | --- | --- | --- | --- | --- | --- | --- |
| **P2** | 0.11 (0.02-0.43) |  |  |  |  |  |  |  |
| **P3** | 0.38 (0.04-0.70) | 0.36 (0.03-0.69) |  |  |  |  |  |  |
| **P4** | 0.28 (0.04-0.60) | 0.49 (0.16-0.81) | 0.42 (0.08-0.74) |  |  |  |  |  |
| **P5** | 0.03 (0.03-0.29) | 0.04 ((-0.3)-0.28) | 0.07 ((-0.2)-0.40) | 0.01 ((-0.3)-0.32) |  |  |  |  |
| **P6** | 0.11 (0.02-0.43) | 0.25 (0.07-0.57) | 0.21 (0.11-0.53) | 0.20 (0.12-0.52) | 0.44 (0.07-0.11) |  |  |  |
| **P7** | 0.06 (0.02-0.39) | 0.16 (0.01-0.48) | 0.20 (0.12-0.52) | 0.44 (0.11-0.76) | 0.07 ((-0.3)-0.25) | 0.05 ((-0.3)-0.27) |  |  |
| **P8** | 0.45 (0.12-0.77) | 0.43 (0.10-0.75) | 0.23 (0.09-0.55) | 0.48 (0.15-0.80) | 0.04 ((-0.2)-0.36) | 0.15 (0.01-0.47) | 0.15 (0.01-0.47) |  |
| **P9** | 0.11 (0.02-0.43) | 0.07 (0.03-0.25) | 0.04 ((-0.2)-0.36) | 0.19 (0.05-0.14) | 0.07 ((-0.4)-0.25) | 0.48 (0.08--0.1) | 0.11 (0.04-0.21) | 0.01 ((-0.3)-0.32) |
|  | **P1** | **P2** | **P3** | **P4** | **P5** | **P6** | **P7** | **P8** |

## **Table S12: Deep squamous eddies**

| **P1** |  |  |  |  |  |  |  |  |
| --- | --- | --- | --- | --- | --- | --- | --- | --- |
| **P2** | 0.53 (0.19-0.85) |  |  |  |  |  |  |  |
| **P3** | 0.27 (0.05-0.59) | 0.36 (0.02-0.68) |  |  |  |  |  |  |
| **P4** | 0.60 (0.30-0.96) | 0.36 (0.02-0.68) | 0.47 (0.14-0.79) |  |  |  |  |  |
| **P5** | 0.53 (0.19-0.85) | 0.27 (0.05-0.59) | 0.36 (0.02-0.68) | 0.07 (0.04-0.25) |  |  |  |  |
| **P6** | 0.53 (0.19-0.85) | 0.60 (0.30-0.96) | 0.36 (0.02-0.68) | 0.36 (0.02-0.68) | 0.27 (0.05-0.59) |  |  |  |
| **P7** | 0.27 (0.05-0.59) | 0.36 (0.02-0.68) | 0.47 (0.14-0.79) | 0.47 (0.14-0.79) | 0.07 (0.04-0.25) | 0.36 (0.02-0.68) |  |  |
| **P8** | 0.54 (0.20-0.86) | 0.37 (0.03-0.69) | 0.16 (0.01-0.48) | 0.16 (0.01-0.48) | 0.37 (0.03-0.69) | 0.37 (0.03-0.69) | 0.16 (0.01-0.48) |  |
| **P9** | 0.36 (0.02-0.68) | 0.47 (0.14-0.79) | 0.60 (0.32-0.97) | 0.60 (0.32-0.97) | 0.06 (0.03-0.26) | 0.47 (0.14-0.79) | 0.60 (0.32-0.97) | 0.21 (0.11-0.53) |
|  | **P1** | **P2** | **P3** | **P4** | **P5** | **P6** | **P7** | **P8** |

## **Table S13: Cobblestone appearance**

| **P1** |  |  |  |  |  |  |  |  |
| --- | --- | --- | --- | --- | --- | --- | --- | --- |
| **P2** | 0.36 (0.03-0.68) |  |  |  |  |  |  |  |
| **P3** | 0.35 (0.01-0.67) | 0.13 (0.02-0.45) |  |  |  |  |  |  |
| **P4** | 0.13 ((-0.2)-0.45) | 0.40 (0.07-0.72) | 0.10 (0.02-0.42) |  |  |  |  |  |
| **P5** | 0.21 (0.11-0.54) | 0.61 (0.29-0.95) | 0.20 (0.12-0.52) | 0.55 (0.22-0.87) |  |  |  |  |
| **P6** | 0.41 (0.08-0.73) | 0.15 (0.01-0.47) | 0.17 (0.15-0.49) | 0.33 (0.01-0.65) | 0.30 (0.02-0.62) |  |  |  |
| **P7** | 0.61 (0.28-0.94) | 0.36 (0.02-0.68) | 0.33 (0.01-0.65) | 0.35 (0.02-0.67) | 0.45 (0.12-0.77) | 0.56 (0.22-0.88) |  |  |
| **P8** | 0.52 (0.19-0.84) | 0.41 (0.08-0.73) | 0.38 (0.04-0.70) | 0.28 (0.04-0.60) | 0.25 (0.07-0.57) | 0.46 (0.13-0.78) | 0.36 (0.02-0.68) |  |
| **P9** | 0.53 (0.28-0.67) | 0.44 (0.07-0.71) | 0.34 (0.06-0.44) | 0.14 ((-0.4)-0.18) | 0.21 (0.05-0.31) | 0.49 (0.08-0.59) | 0.40 (0.27-0.57) | 0.44 (0.27-0.56) |
|  | **P1** | **P2** | **P3** | **P4** | **P5** | **P6** | **P7** | **P8** |

## **Table S14: Elongated and / or anastomosing rete ridges**

| **P1** |  |  |  |  |  |  |  |  |
| --- | --- | --- | --- | --- | --- | --- | --- | --- |
| **P2** | 0.34 (0.01-0.66) |  |  |  |  |  |  |  |
| **P3** | 0.04 (0.03-0.28) | 0.05 (0.03-0.27) |  |  |  |  |  |  |
| **P4** | 0.05 (0.03-0.27) | 0.04 (0.02-0.36) | 0.36 (0.03-0.69) |  |  |  |  |  |
| **P5** | 0.13 (0.04-0.20) | 0.20 (0.12-0.52) | 0.43 (0.10-0.75) | 0.47 (0.14-0.79) |  |  |  |  |
| **P6** | 0.24 (0.08-0.56) | 0.26 (0.06-0.58) | 0.31 (0.01-0.64) | 0.16 (0.01-0.48) | 0.33 (0.01-0.65) |  |  |  |
| **P7** | 0.34 (0.01-0.66) | 0.52 (0.19-0.84) | 0.30 (0.02-0.62) | 0.15 (0.01-0.47) | 0.20 (0.12-0.52) | 0.69 (0.37-1.03) |  |  |
| **P8** | 0.15 (0.01-0.47) | 0.31 (0.01-0.63) | 0.55 (0.22-0.87) | 0.28 (0.04-0.60) | 0.44 (0.11-0.76) | 0.48 (0.14-0.80) | 0.58 (0.39-1.04) |  |
| **P9** | 0.04 (0.01-0.28) | 0.26 (0.06-0.58) | 0.27 (0.05-0.59) | 0.22 (0.10-0.54) | 0.39 (0.06-0.71) | 0.55 (0.22-0.87) | 0.39 (0.06-0.72) | 0.57 (0.24-0.89) |
|  | **P1** | **P2** | **P3** | **P4** | **P5** | **P6** | **P7** | **P8** |

## **Table S15: Altered cellular alignment**

| **P1** |  |  |  |  |  |  |  |  |
| --- | --- | --- | --- | --- | --- | --- | --- | --- |
| **P2** | 0.01 (-0.3-0.32) |  |  |  |  |  |  |  |
| **P3** | 0.16 (-0.4-0.16) | 0.02 (0.01-0.34) |  |  |  |  |  |  |
| **P4** | 0.01 ((-0.3)-0.32) | 0.18 (0.01-0.50) | 0.49 (0.16-0.81) |  |  |  |  |  |
| **P5** | 0.21 (0.11-0.54) | 0.30 (0.02-0.62) | 0.11 (0.02-0.43) | 0.50 (0.17-0.82) |  |  |  |  |
| **P6** | 0.73 (0.39-1.04) | 0.06 (0.03-0.32) | 0.16 (0.04-0.24) | 0.01 ((-0.3)-0.32) | 0.21 (0.11-0.54) |  |  |  |
| **P7** | 0.07 (0.02-0.39) | 0.47 (0.14-0.79) | 0.13 (0.04-0.20) | 0.16 ((-0.1)-0.48) | 0.13 (0.02-0.45) | 0.07 ((-0.2)-0.39) |  |  |
| **P8** | 0.41 (0.08-0.73) | 0.36 (0.02-0.68) | 0.01 ((-0.3)-0.31) | 0.38 (0.05-0.70) | 0.73 (0.43-1.08) | 0.24 (0.08-0.56) | 0.31 (0.01-0.63) |  |
| **P9** | 0.04 (0.02-0.28) | 0.25 (0.07-0.57) | 0.54 (0.21-0.86) | 0.38 (0.05-0.70) | 0.33 (0.01-0.65) | 0.16 ((-0.4)-0.16) | 0.01 ((-0.3)-0.31) | 0.21 (0.11-0.53) |
|  | **P1** | **P2** | **P3** | **P4** | **P5** | **P6** | **P7** | **P8** |

## **Table S16: Parakeratosis**

| **P1** |  |  |  |  |  |  |  |  |
| --- | --- | --- | --- | --- | --- | --- | --- | --- |
| **P2** | 0.25 (0.07-0.57) |  |  |  |  |  |  |  |
| **P3** | 0.17 (0.01-0.49) | 0.55 (0.22-0.87) |  |  |  |  |  |  |
| **P4** | 0.38 (0.05-0.70) | 0.38 (0.05-0.70) | 0.31 (0.01-0.64) |  |  |  |  |  |
| **P5** | 0.36 (0.03-0.69) | 0.60 (0.26-0.92) | 0.42 (0.08-0.74) | 0.56 (0.22-0.88) |  |  |  |  |
| **P6** | 0.33 (0.01-0.65) | 0.82 (0.48-1.14) | 0.63 (0.29-0.95) | 0.44 (0.11-0.76) | 0.66 (0.33-0.98) |  |  |  |
| **P7** | 0.43 (0.09-0.75) | 0.68 (0.35-1.00) | 0.61 (0.28-0.93) | 0.54 (0.21-0.86) | 0.65 (0.32-0.97) | 0.75 (0.42-1.07) |  |  |
| **P8** | 0.43 (0.09-0.75) | 0.68 (0.35-1.00) | 0.48 (0.14-0.80) | 0.43 (0.10-0.75) | 0.65 (0.32-0.97) | 0.81 (0.54-1.20) | 0.74 (0.41-1.06) |  |
| **P9** | 0.48 (0.14-0.80) | 0.61 (0.28-0.93) | 0.53 (0.20-0.85) | 0.48 (0.15-0.80) | 0.47 (0.14-0.79) | 0.55 (0.22-0.87) | 0.80 (0.47-1.12) | 0.66 (0.33-0.99) |
|  | **P1** | **P2** | **P3** | **P4** | **P5** | **P6** | **P7** | **P8** |
