# Supplementary figures and images for: Histological interpretation of differentiated vulvar intraepithelial neoplasia (dVIN) remains challenging—observations from a bi-national ring-study
Source: Virchows Arch. 2021 Mar 8;479(2):305–15. doi: 10.1007/s00428-021-03070-0 (PMC8364542; doi:10.1007/s00428-021-03070-0)

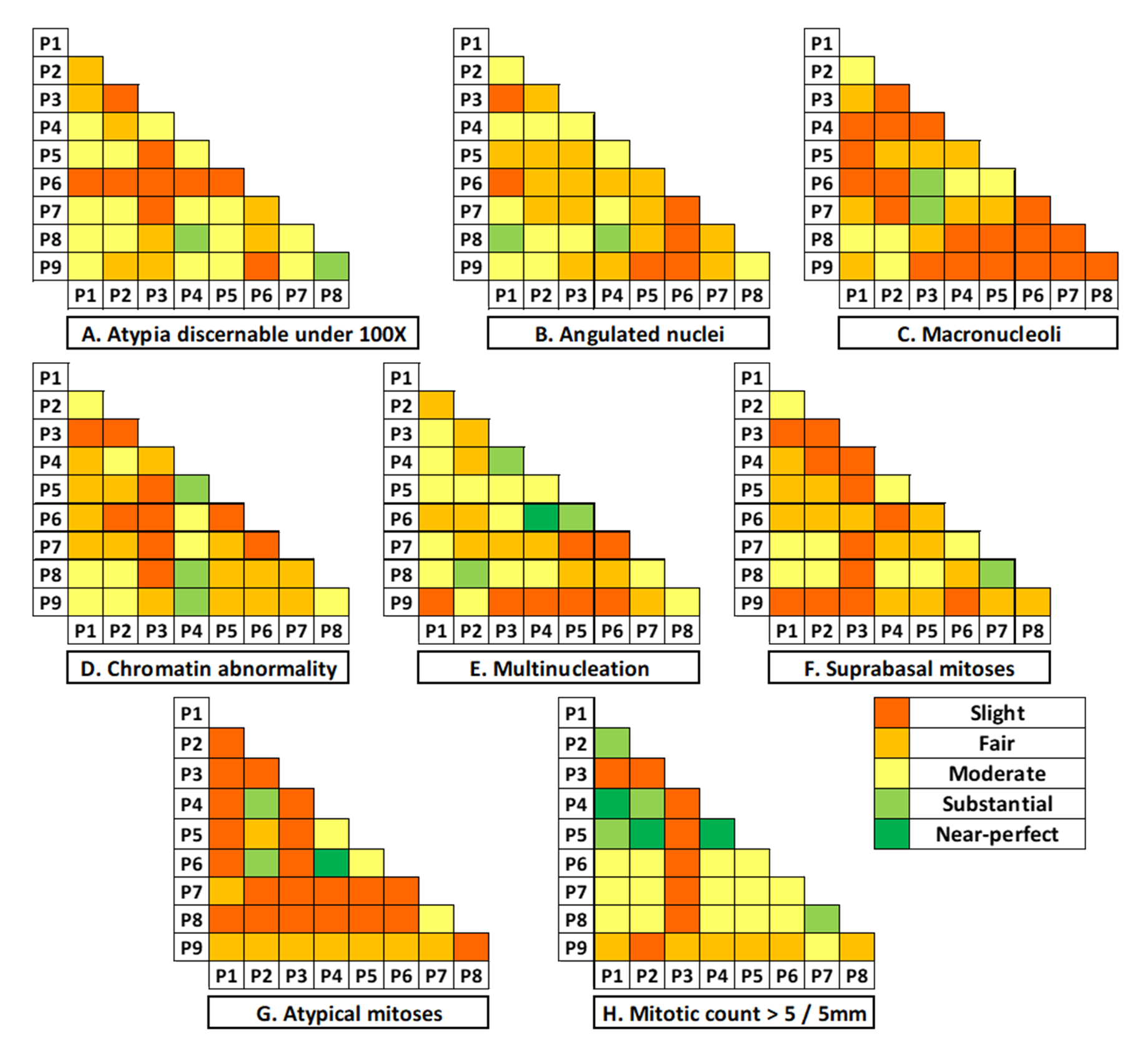

Supplement: Supplementary file 4 — Heat maps depicting the levels of agreement between the participant pairs for the features of nuclear atypia; color-coding corresponds to the levels of agreement (PNG 376 kb) [file 428_2021_3070_Fig6_ESM.png]

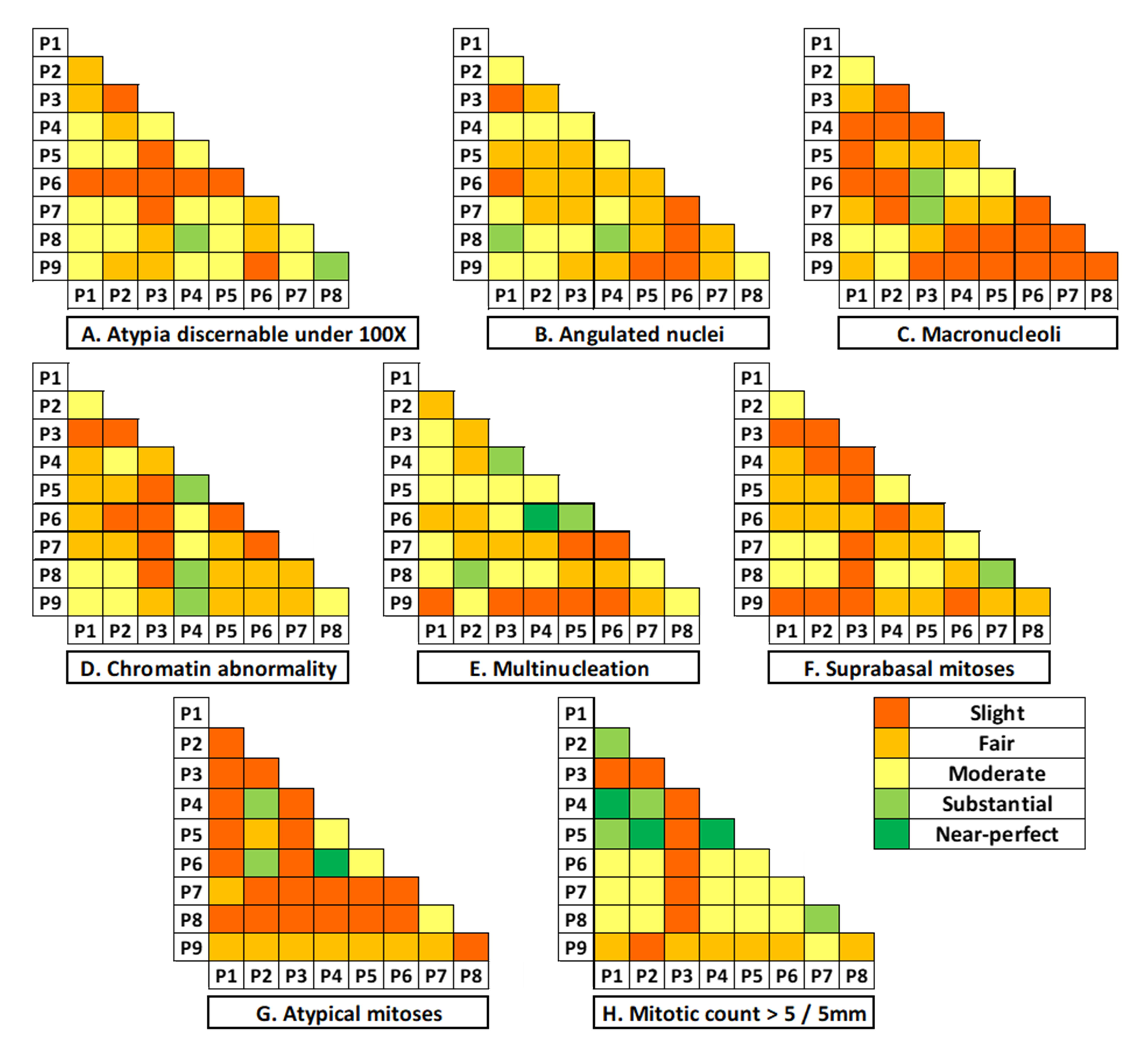

Supplement: Supplementary file 5 — High Resolution Image (TIF 14061 kb) [file 428_2021_3070_MOESM4_ESM.tif]

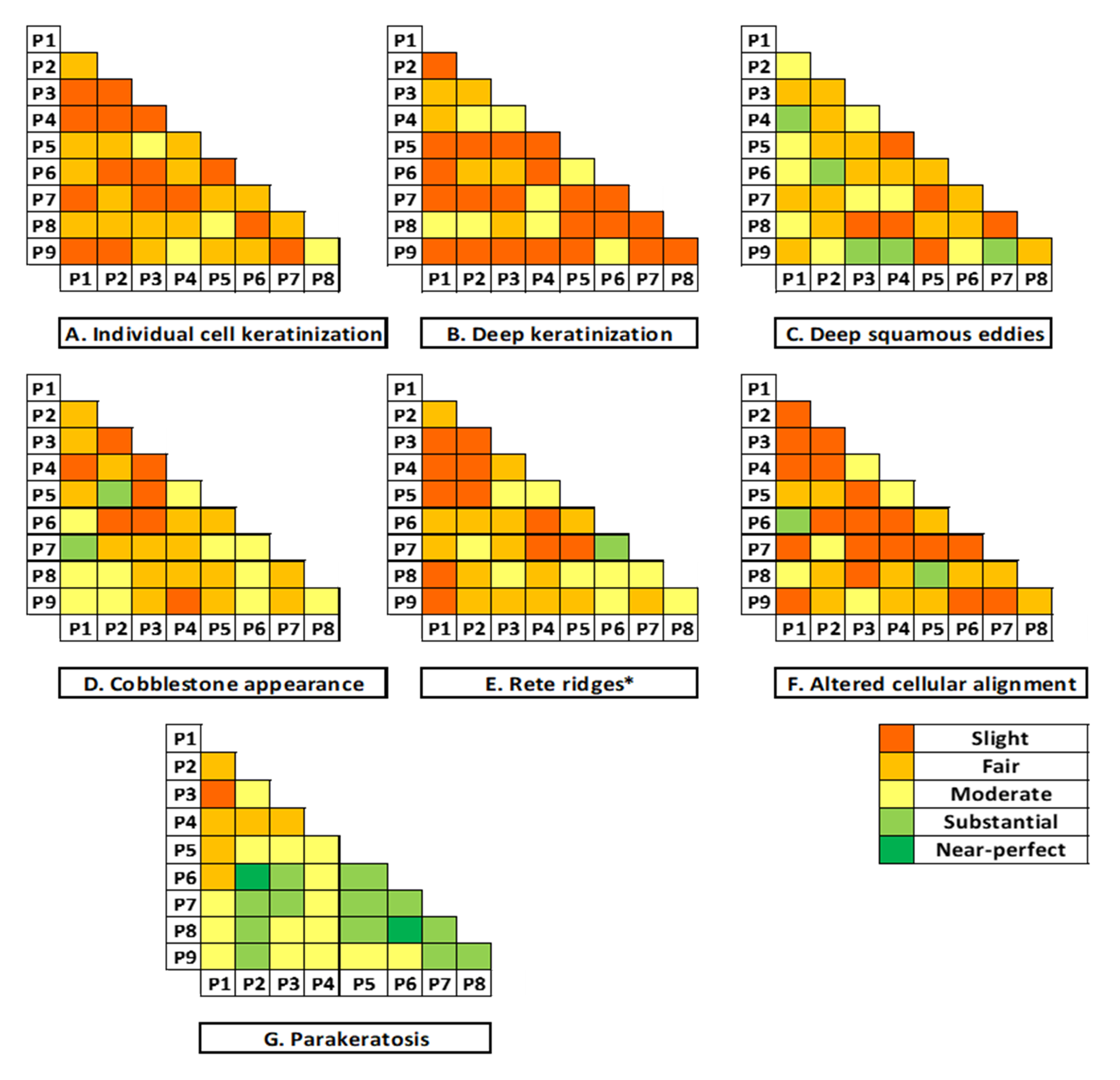

Supplement: Supplementary file 6 — Heat maps depicting the levels of agreement between the participant pairs for the features of disturbed maturation / architecture; color-coding corresponds to the levels of agreement; *elongated and / or anastomosing rete ridges (PNG 499 kb) [file 428_2021_3070_Fig7_ESM.png]

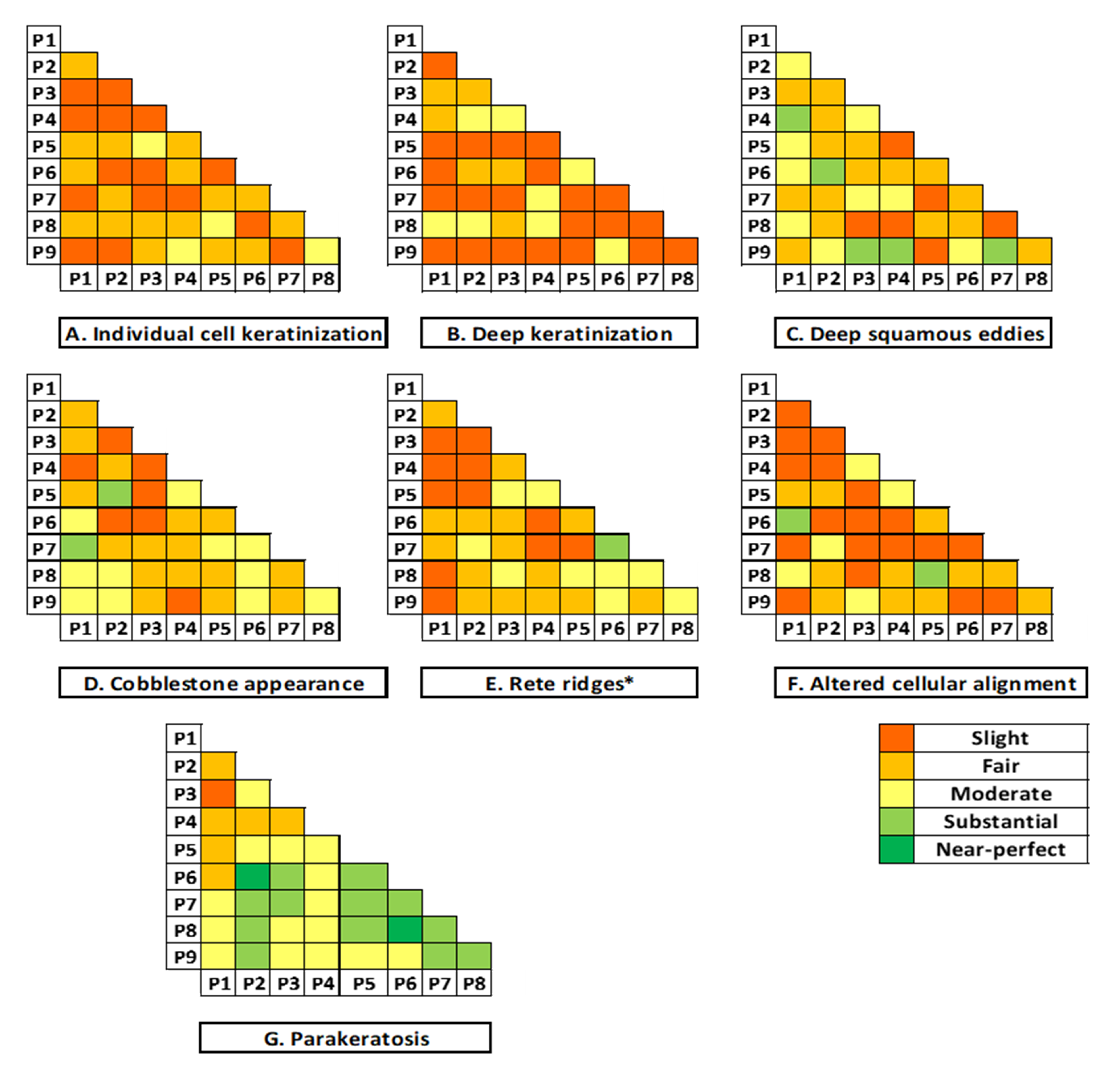

Supplement: Supplementary file 7 — High Resolution Image (TIF 16.3 mb) [file 428_2021_3070_MOESM5_ESM.tif]
